# Editorial: Thyroid hormone actions in cancer

**DOI:** 10.3389/fendo.2023.1219871

**Published:** 2023-05-25

**Authors:** Florencia Cayrol, Helena Andrea Sterle, Maria Del Mar Montesinos

**Affiliations:** ^1^ Laboratorio de Neuroinmunomodulación y Oncología Molecular, Instituto de Investigaciones Biomédicas (BIOMED)-Consejo Nacional de Investigaciones Científicas y Técnicas (CONICET)-Universidad Católica Argentina (UCA), Buenos Aires, Argentina; ^2^ Centro de Investigaciones en Bioquímica Clínica e Inmunología - Consejo Nacional de Investigaciones Científicas y Técnicas (CIBICI-CONICET), Departamento de Bioquímica Clínica, Facultad de Ciencias Químicas, Universidad Nacional de Córdoba, Cordoba, Argentina

**Keywords:** thyroid hormone receptor, thyroid status, integrin alpha V beta 3, cancer progression, antitumor immunity, deiodinases

Thyroid hormones (THs) are well known for being key players in controlling cell metabolism, growth, and many other important physiological processes such as embryonic development and cell differentiation (1). THs exert their biological actions primarily through the binding of 3,5,3′-triiodo-l-thyronine (T3) to its nuclear receptor TR that subsequently homodimerizes (TR/TR) and/or heterodimerizes with retinoid X receptor –RXR- (TR/RXR) to regulate the transcription of target genes containing a TH-response element (TRE). Circulating TH levels are largely stable, in humans approximately 80% of serum T3 is derived from T4 by the iodothyronine deiodinases that are expressed in a tissue-specific manner in fetal and adult life. Importantly, deiodinase-mediated mechanisms within target cells can modulate TH signaling (2). In addition, it was described that both, T3 and T4 (l-thyroxine), activate membrane-bound receptors such as the integrin αvβ3 triggering different signaling pathways that ultimately modulate transcription of a complementary set of genes (3). T4 is the main form of TH produced by the thyroid gland, but it is only minimally active. In the last decades, scientific research has supported a relationship between THs and the pathophysiology of different cancer types (4, 5). In this sense, abnormal THs levels caused by thyroid dysfunction, the disruption of deiodinases, and TH nuclear or membrane receptors’ activity can affect tumor behavior. THs direct actions on cancer cells through genomic and non-genomic pathways can regulate malignant proliferation, differentiation, apoptosis, invasiveness, and angiogenesis in solid (6–8) and hematological (9–11) malignancies. In addition, local control of THs signaling provided by the regulation of deiodinase activity is associated with cancer development, progression, and recurrence by impacting virtually all the hallmarks of cancer (4, 12). Besides, alterations in deiodinase expression and activity are frequent in tumors (13). On the other hand, increasing evidence suggests that THs are modulators of the immune responses, including antitumor immunity, thus influencing tumor progression (14–17). Furthermore, the dysregulation of the thyroid status was also shown to affect the development, progression, and outcomes in cancer patients (18). In addition, some authors propose that tumor cells gain the ability to affect local and systemic homeostasis, including thyroid status, by the release of neurohormonal and immune mediators, that can control the main neuroendocrine centers such as the hypothalamus, pituitary, adrenals, and thyroid (19).

The knowledge of the mechanisms of THs actions in cancer could lead to the identification of new therapeutic targets or to the use of the modulation of the thyroid status to improve treatment outcomes in patients. The overall aim of this Research Topic entitled “*Thyroid hormone actions in cancer*” includes the contribution and significance of TH´s direct actions on tumor cell proliferation, survival, and dissemination; and the association of thyroid status with the risk and progression of oncologic pathologies. This Research Topic consists of two original research articles, one review, and one systematic review. These articles include the study of hepatocellular carcinoma, glioblastoma, thyroid cancer, and pituitary adenoma as the research oncologic models.

In the first original paper Godugu et al., evaluated whether TH analogs have a role in efferocytosis, a process by which apoptotic cells are removed by phagocytic cells. Using *in vitro* and *in vivo* glioblastoma models authors examined how the apoptosis induced by the hormone analog tetraiodothyroacetic acid (tetrac) and chemically modified tetrac actions initiated at the integrin αvβ3 on tumor cells, contribute to the clearance of apoptotic cells. In the other original research article, Lu et al. used Mendelian randomization (MR) analysis to estimate the causal effect of hypothyroidism on the risk for hepatocellular carcinoma (HCC). Single-nucleotide polymorphisms (SNPs) associated with hypothyroidism were screened *via* a genome-wide association study (GWAS). They found that hypothyroidism has a protective causal association with HCC. On the other hand, Wang et al. performed a systematic review of related original studies with the purpose of comprehensively evaluating the relationships between thyroid-related hormones and the risk of thyroid cancer (TC). The authors found a significant association between thyroid-related hormones (TSH, free T4, free T3) and the risk of TC in this study, but further research is needed to understand the underlying mechanisms. Finally, Li et al. showed a rare case of pituitary adenoma producing both GH and TSH simultaneously, which is associated with hyperthyroidism and acromegaly.

## Author contributions

FC: writing–original draft, writing–review and editing. HS: writing–review and editing. MM: writing–review and editing. All authors contributed to the article and approved the submitted version.
